# Pathobiology and transmission of highly and low pathogenic avian influenza viruses in European quail (*Coturnix c. coturnix*)

**DOI:** 10.1186/1297-9716-44-23

**Published:** 2013-03-28

**Authors:** Kateri Bertran, Roser Dolz, Núria Busquets, Virginia Gamino, Júlia Vergara-Alert, Aida J Chaves, Antonio Ramis, Xavier F Abad, Ursula Höfle, Natàlia Majó

**Affiliations:** 1 Centre de Recerca en Sanitat Animal (CReSA), UAB-IRTA, Campus de la Universitat Autònoma de Barcelona, Bellaterra, (Cerdanyola del Vallès), 08193, Spain; 2 Instituto de Investigación en Recursos Cinegéticos, IREC (CSIC, UCLM, JCCM), Ronda de Toledo s/n, Ciudad Real, 13071, Spain; 3 Departament de Sanitat i Anatomia Animals, Universitat Autònoma de Barcelona, Bellaterra, (Cerdanyola del Vallès), 08193, Spain

## Abstract

European quail (*Coturnix c. coturnix*) may share with Japanese quail (*Coturnix c. japonica*) its potential as an intermediate host and reservoir of avian influenza viruses (AIV). To elucidate this question, European quail were experimentally challenged with two highly pathogenic AIV (HPAIV) (H7N1/HP and H5N1/HP) and one low pathogenic AIV (LPAIV) (H7N2/LP). Contact animals were also used to assess the viral transmission among birds. Severe neurological signs and mortality rates of 67% (H7N1/HP) and 92% (H5N1/HP) were observed. Although histopathological findings were present in both HPAIV-infected groups, H5N1/HP-quail displayed a broader viral antigen distribution and extent of microscopic lesions. Neither clinical nor pathological involvement was observed in LPAIV-infected quail. Consistent long-term viral shedding and effective transmission to naive quail was demonstrated for the three studied AIV. Drinking water arose as a possible transmission route and feathers as a potential origin of HPAIV dissemination. The present study demonstrates that European quail may play a major role in AI epidemiology, highlighting the need to further understand its putative role as an intermediate host for avian/mammalian reassortant viruses.

## Introduction

Avian influenza (AI) represents a major disease problem, not only for poultry but also for other avian species, mammals, and human beings [1]. The constant outbreaks detected around the world in poultry and wild birds are of concern to the economics of the poultry industry, to wildlife conservation, and to animal and public health [2]. Susceptibility to AI viruses (AIV) varies deeply among wild bird and poultry species, as well as their possible role as sentinels, intermediate hosts or reservoirs. Gallinaceous poultry are considered to be highly susceptible [3,4], whereas waterfowl have long been recognized as natural reservoirs, although they may show variable morbidity depending on the infective viral strain [5-7].

Since the first reported case of AI in Japanese quail (*Coturnix c. japonica*) in Italy (1966–1968) [8], influenza viruses of several subtypes have been isolated from quail in North America, Europe, and Asia through periodic surveillance and sporadic outbreaks [9-11]. Several experimental infections in Japanese quail have reported either higher, similar or lower susceptibilities than chickens to H5 highly pathogenic AIV (HPAIV) [3,12-15]. Moreover, inoculation of low pathogenic AIV (LPAIV) representing subtypes H1 to H15 proved that Japanese quail may support the replication (predominantly in the respiratory tract) of almost all of them [16]. More recently, several studies suggested that multiple in vivo passages in Japanese quail facilitate the adaptation of duck AIV to chicken [17-20]. These cumulative observations along the years have been recently explained by molecular adaptation of quail AI strains, especially in hemagglutinin (HA) and neuraminidase genes, which amino acids pattern might be intermediate between those of duck and chicken viruses [21-23]. In addition, quail carry sialic acid receptors functional for binding of avian and human influenza viruses [24,25]. Therefore, Japanese quail may provide an optimal environment for the adaptation of wild bird AIV, generating novel variants that can cross the species barrier to domestic poultry and human beings. Surprisingly, the epidemiological significance observed for the Japanese quail has not yet been demonstrated for the European quail (*Coturnix c. coturnix*).

The European quail, also called common or wild quail, is a partial migrant whose breeding range extends from the Atlantic to Lake Baikal and from the Arctic Circle to the tropics [26]. A decline in the number of European quail in the Western Palearctic over the past few decades has stimulated the release of Japanese quail as game birds in various European countries, leading to hybridization between both species in the field [27]. Even though European and Japanese quail show a high overall similarity in morphological, behavioral, and ecological features that made some authors conclude that they belong to the same species [28], they are distinguishable by characteristic morphological traits and calls [27,29]. The Japanese quail, also called domestic quail, is found in the wild in Asia [26], but is best known in its domestic form in Europe, Asia, North America, and India where it is generally ranged in outdoor game farms for restocking and hunting purposes [30] as well as for meat and egg production [31]. Particularly in Spain, Japanese and European quail currently comprise 4.7% of the global avian meat production system [32] with an ever-increasing population size along the years. Considering that open range rearing of birds has been identified as one of the factors contributing to the increase of AI outbreaks and their effect [2], specific attention should be paid to the quail, which may have a role in the spread and exacerbation of the disease.

To date, various studies have assessed the susceptibility of Japanese quail to H5 HPAIV and LPAIV [3,12-16]. However, the present study is the first experimental infection investigating the susceptibility of European quail to AIV. On the one hand, it is the first attempt to assess the potential viral shedding of HPAIV and LPAIV in this species, and the likelihood of effective transmission among quail. On the other hand, it represents a comparative study of the pathogenesis and viral distribution in tissues of two different HPAIV subtypes (H7 and H5). The overall results depict the role that European quail may play in the epidemiology of AI, and its putative responsibility in an interspecies outbreak.

## Materials and methods

### Viruses

Three strains of AIV were used: H7 HPAIV, H5 HPAIV, and H7 LPAIV. The H7 HPAIV [A/Chicken/Italy/5093/1999 (H7N1) (H7N1/HP)] was isolated during the 1999–2000 Italian epidemic [33]. The H5 HPAIV [A/Great crested grebe/Basque Country/06.03249/2006 (H5N1) (H5N1/HP)] was obtained from the only reported case of H5N1 HPAIV in wild birds in Spain so far [34]. The H7 LPAIV [A/*Anas platyrhynchos*/Spain/1877/2009 (H7N2) (H7N2/LP)] was obtained from the ongoing surveillance program carried out in Catalonia (Northeast Spain). The deduced amino acid sequence of the region coding for the cleavage site of the precursor of the HA molecule were PEIPKGSRVRR*GLF for the H7N1/HP and PEIPKGR*GLF for the H7N2/LP, being typical of HPAIV and LPAIV, respectively [35].

Virus stocks were produced in specific pathogen free (SPF) chicken eggs. The allantoic fluids were harvested at 48 hours post-inoculation (hpi) (H7N1/HP and H5N1/HP) and 72 hpi (H7N2/LP). Viruses were tenfold diluted in phosphate buffer saline (PBS) for titration in 9-day-old embryonating SPF chicken eggs. The mean embryo lethal dose (ELD_50_) and the mean embryo infectious dose (EID_50_) for the HPAIV and LPAIV isolates, respectively, were determined [36].

### Animals

European quail (Urgasa S.A., Lleida, Spain) of approximately two months of age were used in this study. Male and female birds were included in almost equal numbers. Before the infection, serum samples of all individuals were confirmed to be seronegative for AIV by a competition ELISA test (C-ELISA) (IDVET, Montpellier, France). Furthermore, oropharyngeal (OS) and cloacal (CS) swabs were ensured to be negative for AIV by real time RT-PCR (RRT-PCR). Each experimental group was housed in a different negative pressured isolator with HEPA-filtered air in the animal biosafety level 3 (ABSL-3) facilities of *Centre de Recerca en Sanitat Animal* (CReSA). Quail were kept one week for acclimation, and feed and water were provided *ad libitum* throughout the experiment. All procedures were performed according to the requirements of the Ethical Commission of Animal Experimentation of the Autonomous Government of Catalonia.

### Experimental design

Eighty birds were randomly separated into seven groups: six challenged groups with 12 birds/group and one control group with 8 birds (Table 1). For each virus, quail were subdivided into two experimental groups, A and B (*n* = 12/group). Groups 1A, 2A, and 3A were used to evaluate morbidity, mortality, transmissibility, and viral shedding pattern. Groups 1B, 2B, and 3B were used for the pathological studies. All animals were inoculated intranasally with 10^6^ EID_50_ (for the LPAIV) or 10^6^ ELD_50_ (for the HPAIV) of the corresponding challenge virus in a volume of 0.5 mL, except four birds of each A group which were used as contact animals. Contact birds were placed into the isolators four hours after inoculating the other birds and after changing drinking water. A seventh group (group C) (*n* = 8) was used as negative controls; these quail were inoculated intranasally with PBS solution. Amounts of virus were verified by performing a RRT-PCR of both the original non-diluted viruses and the inocula.

**Table 1:** Experimental design of the study

**Group**	**Inoculum**	**Titer**	**No. animals**
1A	H7N2/LP	10^6^ EID_50_	12 (8+4)*
1B	H7N2/LP	10^6^ EID_50_	12
2A	H7N1/HP	10^6^ ELD_50_	12 (8+4)*
2B	H7N1/HP	10^6^ ELD_50_	12
3A	H5N1/HP	10^6^ ELD_50_	12 (8+4)*
3B	H5N1/HP	10^6^ ELD_50_	12
C	PBS	-	8

*In A groups, eight quail were inoculated and four quail were left as contact birds.

H7N2/LP, A/*Anas platyrhynchos*/Spain/1877/2009; H7N1/HP, A/Chicken/Italy/5093/1999; H5N1/HP, A/Great crested grebe/Basque Country/06.03249/2006; ELD_50_, mean embryo lethal dose; EID_50_, mean embryo infectious dose; PBS, phosphate buffer saline.

### Sampling

All birds were monitored daily for clinical signs. During the first 10 days post-inoculation (dpi), at 12 dpi, and 15 dpi, OS, CS and feather pulp (FP) samples were obtained from quail from the A groups to measure viral shedding by RRT-PCR. Drinking water was collected with a 1 mL syringe at the same time points, and it was changed on a daily basis. The same samples were collected from group C. Mortality and mean death times (MDT) were calculated from the A groups. At 3, 5, 8, and 15 dpi, three animals from groups B and two animals from group C were euthanized using intravenous sodium pentobarbital (100 mg/kg, Dolethal®, Vétoquinol, Cedex, France). Surviving birds were euthanized at the end of the experiment (15 dpi). Blood samples were collected before euthanasia to detect AI antibodies by C-ELISA testing. As it was terminal, bleeding was done from the heart after previous anesthesia with intramuscular injection of ketamine/xylazine (10 g/kg body weight, Imalgene® 1000 and 1 g/kg body weight, Xilagesic® 2%). All euthanized and naturally dead quail from the B groups were necropsied to evaluate gross lesions and obtain samples for histopathological studies. Swabs and FP samples were placed in 0.5 mL of Dulbecco’s Modified Eagle’s Medium (DMEM) (BioWhittaker®, Lonza, Verviers, Belgium) with 600 μg/mL penicillin and streptomycin. These samples, together with drinking water samples and serum samples, were stored at −80 °C until further use.

### Pathologic examination and immunohistochemical testing

Necropsies and tissue sampling were performed according to standard protocols [37]. After fixation in 10% neutral buffered formalin and embedding in paraffin, tissue sections were processed routinely for hematoxylin/eosin (HE) staining. The following tissues were examined: esophagus, crop, proventriculus, gizzard, duodenum, jejunum-ileum, cecum/cecal tonsil, colon, rectum, pancreas, liver, kidney, adrenal gland, gonad, nasal turbinates, trachea, lung, heart, breast muscle, skin, bone marrow, spleen, bursa of Fabricius, thymus, brain, spinal cord, and sciatic nerve. In addition, an immunohistochemical (IHC) technique was performed as previously described [38,39]. The primary antibody was a mouse-derived monoclonal commercial antibody against nucleoprotein (NP) of influenza A virus (IgG2a, Hb65, ATCC). As a secondary antibody, a biotinylated goat anti-mouse IgG antibody (GaMb, Dako E0433, Glostrup, Denmark) was used. Tissues previously demonstrated to be positive against NP of influenza A virus by IHC were used as a positive control. Duplicated samples of all animals incubated without the primary antibody, as well as tissues from sham-inoculated animals processed as usual by IHC, served as negative controls. The following score was used to grade the staining in the tissues: no positive cells (−), single positive cells (+), scattered groups of positive cells (++), widespread positivity (+++).

### Viral RNA detection by RRT-PCR

Viral RNA from OS, CS, FP, and drinking water samples was extracted with NucleoSpin® RNA virus kit (Macherey-Nagel, Düren, Germany) following the manufacturer’s instructions. The resulting viral RNA extracts were tested by one-step RRT-PCR for the detection of a highly conserved region of the matrix (*M*) gene in Fast7500 equipment (Applied Biosystems, Foster City, CA, USA) using the primers and probe previously described [40] and the amplification conditions described by Busquets et al. [41]. Samples with a threshold cycle (Ct) value ≤ 40 were considered positive for influenza A viral RNA. Viral shedding was analyzed by ANOVA test for significant differences (*p* < 0.05) using the Statistical Package for the Social Sciences for Windows Version 20.0.

### Serology

A C-ELISA test was carried out to detect antibodies against the NP of AIV using the commercially available kit ID Screen® Influenza A Antibody Competition (IDVET, Montpellier, France), according to the manufacturer’s instructions. In addition, a hemagglutination inhibition (HI) test was performed to titrate antibodies against specific H5- (in H5N1/HP serum samples) and H7- (in H7N2/LP and H7N1/HP serum samples) subtypes. The HI assays were performed according to standard procedures [42] with chicken red blood cells and commercial inactivated H5- and H7-antigens (GD-Deventer, The Netherlands). To avoid nonspecific positive reactions, sera were pre-treated by adsorption with 10% chicken red blood cells. Titers were expressed as geometric mean titers (GMT-log_2_); GMT of 3 log_2_ or greater were considered positive. Previously known positive and negative sera were used as controls.

## Results

### Morbidity and mortality

**Figure 1:**
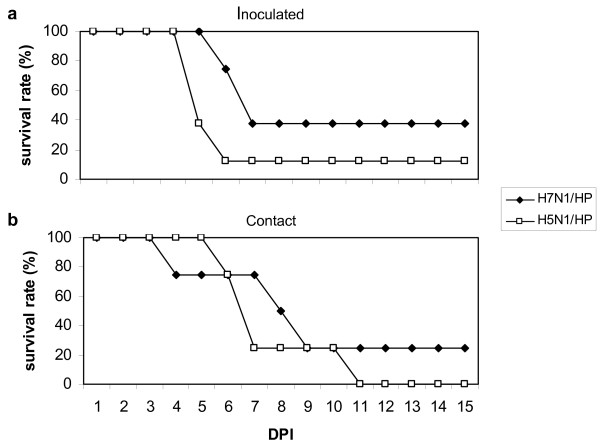
**Survival rates of quail intranasally challenged with either H7N1/HP or H5N1/HP. a.** Intranasally inoculated quail. **b.** Contact quail.

Clinical signs and mortality were only observed in HPAIV-infected groups (groups 2 and 3) and were similar between inoculated and contact birds. Some of the quail (17% H7N1/HP-challenged and 58% H5N1/HP-challenged animals) displayed nonspecific clinical signs, consisting of lethargy, anorexia, and ruffled feathers, that progressed to death or severe neurological signs (e.g., incoordination, torticollis, circling, head tremors, head tilt, and opisthotonus) within 24 h. The onset times of these nonspecific signs were 6 dpi for H7N1/HP-group and 4 dpi for H5N1/HP-group. Two H7N1/HP-challenged quail (17%) and three H5N1/HP-challenged quail (25%) presented an acute fatal progression of the infection, displaying neurological signs without previous nonspecific signs at 7 dpi and 5 dpi, respectively. However, in other cases (33% in H7N1/HP-group and 8% in H5N1/HP-group) quail were found dead without previous clinical signs. Only one bird, belonging to the H5N1/HP-group, recovered after showing nonspecific clinical signs at 6–7 dpi. All animals with neurological signs, recumbent or both were euthanized for ethical reasons. The survival rates and the MDT of the HPAIV-infected groups (groups 2 and 3) throughout the experiment are summarized in Figure 1 and Table 2.

**Table 2:** Survival rates and MDT of quail intranasally challenged with either H7N1/HP or H5N1/HP

**Virus**	Mortality*		
	Inoculated (MDT)	Contact (MDT)	Total
H7N1/HP	63% (6.6)	75% (7)	67%
H5N1/HP	88% (5.3)	100% (6)	92%

*#dead/total X 100.

MDT, mean death time (dpi); H7N1/HP, A/Chicken/Italy/5093/1999; H5N1/HP, A/Great crested grebe/Basque Country/06.03249/2006.

### Gross findings

Consistent gross lesions were only observed in HPAIV-infected groups (groups 2 and 3) and were similar between inoculated and contact birds. At 3 dpi, one H7N1/HP-quail (group 2) presented multifocal petechia on the proventriculus-gizzard junction mucosa. However, the foremost lesions in the H7N1/HP-group were observed at 5 dpi, which consisted of moderate splenomegaly with pallor or parenchymal mottling and pancreatic lesions characterized by multifocal necrotic areas of 1 mm-diameter. Lesions in H5N1/HP-quail (group 3) were most pronounced and were detected throughout the experiment in all necropsied birds. At 3 dpi, liver pallor in one bird was observed. The quail found dead at 4 dpi presented spleen pallor and multifocal areas in the pancreas. Such pancreatic lesion, as well as thymus atrophy, was observed until the end of the experiment in all necropsied birds. At 5 dpi, spleen pallor was observed in one bird. No gross lesions were observed in H7N2/LP-infected birds (group 1) or in birds from the control group (group C).

### Histopathological findings

**Figure 2:**
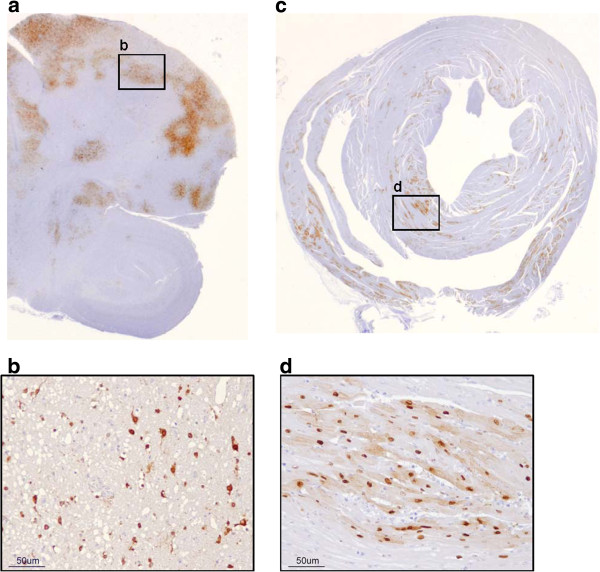
**Distribution of NP antigen in positive tissues of a quail intranasally challenged with H5N1/HP. a.** Brain, 7 dpi. **b.** Positive staining in nucleus and cytoplasm of neurons and glial cells. **c**. Heart, 5 dpi. **d**. Positive staining in nucleus and cytoplasm of myocardiocytes.

Histological lesions and influenza A viral NP were only observed in HPAIV-infected quail (groups 2 and 3) (Tables 3, 4). In H7N1/HP-quail, prevailing histological lesions were observed at 5 and 8 dpi mainly in the pancreas, heart, and brain, but also in the gizzard, cecal tonsil, and spinal cord (Table 3). H5N1/HP-challenged birds consistently showed marked lesions in the tissues mentioned for the H7N1/HP-infected quail and also, to a lesser extent, in the rectum, kidney, and skeletal muscle from the breast (Table 4). Accordingly, presence of H5N1/HP in tissues, as determined by IHC, was more intense than H7N1/HP. The most consistent finding, prevalent throughout almost all the experiment within both HPAIV-challenged groups, was moderate to severe multifocal to coalescent lytic necrosis of the acinar epithelium of the pancreas and endothelial activation indicative of acute inflammation. The main findings in the brain consisted of moderate to severe multifocal areas of malacia in the cerebral hemispheres, associated with spongiosis of the neuropil, neuronal chromatolysis, and gliosis (Figures 2a, 2b). Overt severe necrosis of ependymal cells of the ventricles was present in all affected quail. The cerebellum frequently showed multifocal areas of moderate to severe chromatolysis of Purkinje neurons at 3, 5, and 7 dpi of H5N1/HP-infected quail, sometimes associated with non-suppurative perivascular inflammatory infiltrate. The heart was also consistently affected, with multifocal to diffuse myocardial degeneration and necrosis consisting of hyalinization and fragmentation of cardiac myocytes, often associated with mild lymphoplasmacytic infiltrate (Figures 2c, 2d). In general, IHC staining was mainly nuclear and sometimes also cytoplasmic in distribution and correlated well with histopathological findings.

**Table 3:** Average distribution of AIV-NP antigen in positive tissues from quail intranasally challenged with H7N1/HP

Tissue*	**3 dpi**	**5 dpi**	**8 dpi**	**15 dpi**	**Predominant cell types**	**Associated lesion**
Gizzard	–	++	–	–	Epithelial cells of the ventricular glands	Severe multifocal necrosis, mild lymphoplasmacytic infiltrate
Cecal tonsil	–	+	–	–	Epithelial cells of the glands, cells of the muscularis externa	Focal necrosis, mild lymphoplasmacytic infiltrate
Pancreas	–	++	+++	–	Acinar cells, endothelial cells	Severe multifocal to coalescent lytic necrosis, lymphoplasmacytic infiltrate, edema
Nasal turbinates	+	–	–	–	Respiratory epithelial cells	Lymphoplasmacytic infiltrate in lamina propria
Trachea	–	+	–	–	Goblet cells	NSL
Heart	–	++	–	–	Myocardiocytes, endothelial cells	Severe multifocal necrosis, mild lymphoplasmacytic infiltrate
Spleen	–	+	+	–	Endothelial cells, macrophages	NSL
Brain	–	+++	++	–	Neurons, Purkinje cells, ependymal cells, glial cells, endothelial cells	Malacia in cortex, necrosis of ependymal cells of ventricles and epithelial cells of choroid plexus, chromatolysis of Purkinje cells, lymphoplasmacytic infiltrate
Spinal cord	–	+++	–	–	-	Malacia in grey matter, necrosis of the ependyma and neuropil

*Tissues not present appeared overtly normal on histopathological analysis and did not show positive IHC staining.

– = no positive cells; + = single positive cells; ++ = scattered groups of positive cells; +++ = widespread positivity.

dpi, days post-inoculation; NSL, no significant lesions.

**Table 4:** Average distribution of AIV-NP antigen in positive tissues from quail intranasally challenged with H5N1/HP

Tissue*	**3 dpi**	**4 dpi**	**5 dpi**	**6 dpi**	**7 dpi**	**Predominant cell types**	**Associated lesion**
Proventriculus	–	–	–	+	–	Epithelial cells of the proventricular glands, cells of the muscularis externa	Severe multifocal necrosis, mild lymphoplasmacytic infiltrate
Gizzard	–	–	+	++	++	Epithelial cells of the ventricular glands, cells of the muscularis externa	Severe multifocal necrosis, mild lymphoplasmacytic infiltrate
Cecal tonsil	–	–	+	+++	+	Cells of the lamina propria	Mild lymphoplasmacytic infiltrate
Rectum	–	–	–	+	+	Cells of the muscularis externa of the lamina propria	Vacuolation, degeneration, mild lymphoplasmacytic infiltrate
Pancreas	+	–	++	++	+	Acinar cells, endothelial cells	Severe multifocal to coalescent lytic necrosis, lymphoplasmacytic infiltrate, edema
Kidney	+	+	+	+	+++	Collecting tubular epithelial cells, endothelial cells	Moderate to severe necrosis, mild lymphoplasmacytic infiltrate
Adrenal gland	+	–	–	–	–	Corticotrophic and corticotropic cells	NSL
Nasal turbinates	–	++	–	–	–	Respiratory epithelial cells	Lymphoplasmacytic infiltrate in lamina propria
Heart	+	++	+++	++	+++	Myocardiocytes, endothelial cells	Severe multifocal necrosis, mild lymphoplasmacytic infiltrate
Skeletal muscle	–	–	+	++	++	Myocytes, endothelial cells	Moderate multifocal necrosis, mild lymphoplasmacytic infiltrate
Spleen	–	–	–	–	+	Endothelial cells, macrophages	NSL
Brain	++	+++	+++	+++	+++	Neurons, Purkinje cells, ependymal cells, glial cells, endothelial cells	Malacia in cortex, necrosis of ependymal cells of ventricles and epithelial cells of choroid plexus, chromatolysis of Purkinje cells, lymphoplasmacytic infiltrate.

*Tissues not present appeared overtly normal on histopathological analysis and did not show positive IHC staining.

– = no positive cells; + = single positive cells; ++ = scattered groups of positive cells; +++ = widespread positivity.

dpi, days post-inoculation; NSL, no significant lesions.

### Viral RNA detection by RRT-PCR

**Figure 3:**
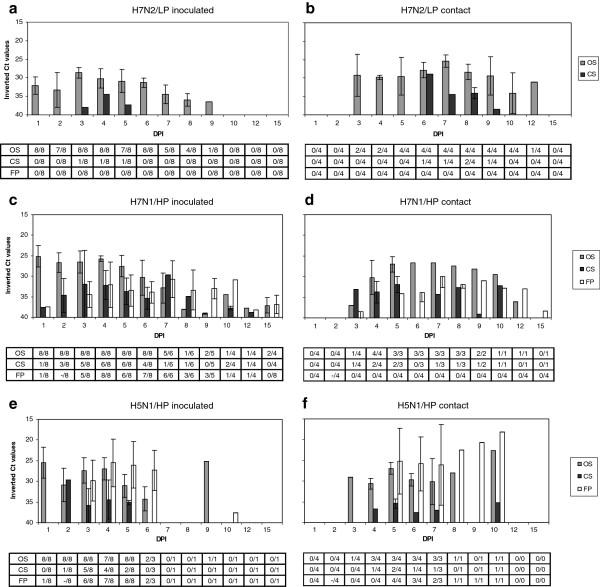
**Viral RNA shedding detected by RRT-PCR in quail experimentally challenged with AIV.** Results are expressed as inverted Ct-values and shown as means of positive individuals ± SD. Tables indicate the ratio between positive quail and total quail examined per day and sample. Ct, cycle of threshold; DPI, days post-inoculation; OS, oropharyngeal swabs; CS, cloacal swabs; FP, feather pulps. **a.** Quail intranasally inoculated with H7N2/LP. **b.** Contact quail of H7N2/LP. **c**. Quail intranasally inoculated with H7N1/HP. **d.** Contact quail of H7N1/HP. **e.** Quail intranasally inoculated with H5N1/HP. **f.** Contact quail of H5N1/HP.

Real time RT-PCR was performed on OS, CS, FP, and drinking water samples of the A groups. Oropharyngeal swabs of H7N2/LP-challenged birds (group 1) tested positive until 9 dpi for inoculated birds peaking at 3 dpi, and until 12 dpi for contact birds peaking at 7 dpi (Figures 3a, 3b). Viral RNA from CS was detected in one animal during 3 days (3–5 dpi) and in two contact animals for 4 days (6–9 dpi). Feather pulp samples tested negative in this H7N2/LP-group. In H7N1/HP-inoculated quail (group 2), viral RNA was detected in all the studied samples (OS, CS, FP) from 1 dpi until before death, although oral shedding was predominant (Figure 3c). Viral RNA detection from contact H7N1/HP-birds was similar to that observed in inoculated quail, although with two days of delay (Figure 3d). For H5N1/HP-inoculated quail (group 3), oral shedding was also higher than cloacal shedding, although FP samples had high amounts of viral RNA as well (Figure 3e). H5N1/HP viral RNA amounts were less homogenous than for H7N1/HP among dpi and types of sample. Contact H5N1/HP-quail had a similar shedding profile to the inoculated ones, although starting two days later (Figure 3f). HPAIV-challenged quail orally shed significantly higher amounts of viral RNA than the LPAIV-challenged quail (*p* < 0.05), especially on 1, 2, and 4 dpi. Moreover, FP from H5N1/HP-challenged quail contained significantly more viral RNA than FP from H7N1/HP-infected quail (*p* < 0.05).

Presence of H7N2/LP viral RNA in drinking water samples coincided with the days where quail’s samples reached maximum viral RNA values (4–6 dpi). H7N1/HP viral RNA was present in water during almost all the experiment (3–15 dpi), being quite stable in time. Existence of H5N1/HP viral RNA in drinking water was manifested at 2 dpi and at 6 dpi, at levels akin to those of H7N1/HP.

### Serology

Before infection, all serum samples tested negative for antibodies against influenza A NP. Almost all the H7N2/LP-inoculated quail (7/8) and all the H7N2/LP-contact quail (4/4) were antibody-positive at 15 dpi, with GMT of 7.9 and 7.3, respectively (Table 5). Besides, all HPAIV-inoculated birds (both H7N1/HP and H5N1/HP) seroconverted from 5 dpi onwards, with GMT steadily increasing until 15 dpi from 4.7 to 7.0 in the case of H7N1/HP-inoculated quail (Table 5).

**Table 5:** Serological data of quail intranasally challenged with either H7N2/LP, H7N1/HP or H5N1/HP

**Group**	**3 dpi**		**5 dpi**		**9 dpi**		**15 dpi**	
	**ELISA**	HI*	**ELISA**	HI*	**ELISA**	HI*	**ELISA**	HI*
H7N2/LP Inoculated	nd	nd	nd	nd	nd	nd	7/8	7/8 (7.9)
H7N2/LP Contact	nd	nd	nd	nd	nd	nd	4/4	4/4 (7.3)
H7N1/HP Inoculated^†^	0/3	0/3	3/3	3/3 (4.7)	3/3	3/3 (6.3)	3/3	3/3 (7.0)
H5N1/HP Inoculated^†^	0/3	0/3	3/3	3/3 (6.0)	nd	nd	nd	nd

dpi, days post-inoculation; ELISA, C-ELISA; HI, hemagglutination inhibition; nd, no data; H7N2/LP, A/*Anas platyrhynchos*/Spain/1877/2009; H7N1/HP, A/Chicken/Italy/5093/1999; H5N1/HP, A/Great crested grebe/Basque Country/06.03249/2006.

*GMT (log_2_) are indicated in parenthesis. GMT include only positive birds.

^†^No data is available for contact quail in these groups.

## Discussion

This is the first experiment which demonstrates that European quail (*Coturnix c. coturnix*) can be infected with both HPAIV and LPAIV. This quail subspecies can be found not only in the wild all over the Palearctic region, but also in many parts of Europe as a game bird species raised in outdoor operations. Despite the relevance of this game bird species, no studies had previously investigated its AIV infection dynamics. The aim of the present study was to elucidate the putative role of European quail in the ecology of influenza A viruses by assessing the pathogenesis, transmissibility, and viral shedding of quail experimentally infected with two different HPAIV subtypes (H7N1 and H5N1) and one LPAIV (H7N2).

Quail exhibited a high susceptibility to both HPAIV used in this study, as demonstrated by severe clinical signs and high mortality rates. With the earliest onset, most rapid progression of disease, and shortest MDT in H5N1/HP-infected quail, it is apparent that this virus is more virulent for this species than the H7N1/HP [43]. The high pathogenicity observed for both HPAIV is in accordance with natural and experimental H5N1 HPAIV infections in chickens and other gallinaceous species, among which are Japanese quail [3,4,14]. Although previous studies with Japanese quail reported minimal clinical signs or even sudden deaths without apparent symptoms [3,14,15], clinically neurological dysfunction was an evident sign in most of the HPAIV-infected quail of the present study. Certain gross findings indicative of AI were not as extensive and obvious as for chickens (e.g., presence of edematous, hemorrhagic, and necrotic cutaneous lesions), but affected tissues were known target organs for influenza A viruses in other gallinaceous species, including Japanese quail [3,4,14,44]. Interestingly, H5N1/HP showed a broader tissue distribution compared with H7N1/HP, suggesting that virus replication in a particular target organ other than respiratory or intestinal organs may contribute to the virulence of the HPAIV in quail, as previously stated [15]. Particularly, neurotropism is considered one of the main factors for the fatal course of AI in birds [45,46], evidenced in our study by the higher virulence of the H5N1/HP compared with H7N1/HP. Our findings in H7N2/LP correlate well with those of Makarova et al. [16], in which a wide range of LPAIV subtypes could replicate efficiently in Japanese quail, predominantly in the respiratory tract. In our study, European quail could also maintain the infection without clinical involvement, and shed the virus mainly orally during a substantial period.

Effective viral transmission from inoculated quail to naive contact birds was confirmed for the three studied viruses, even though their origin avian hosts were as diverse as chicken, mallard, and great crested grebe. This finding suggests that adaptation may not be needed to allow AIV to replicate and transmit in European quail, confirming the substantial role that this species may play in AI epidemiology. As in a previous work with H5N1 HPAIV in Japanese quail [14], both HPAIV used in our study confirmed to be able to transmit among European quail. Moreover, transmission of H7 isolates (both H7N1/HP and H7N2/LP) is of great importance because: I) this is the first transmission evidence of an H7 HPAIV in quail so far; and II) previous experiments with LPAIV failed to confirm this capability in Japanese quail [16]. Not only had the onset of clinical signs proved infection in contact birds, but also their antibody responses (in the case of H7N2/LP challenge) and their efficient viral shedding. Given that quail shed virus mainly orally, contact birds might have been infected by the oral-oral route. In fact, such viral shedding predominance, also stated in previous studies with Japanese quail [14-16], is already known to differ from that observed in LPAIV waterfowl reservoirs [47].

Ingestion of contaminated water has already been suggested as a possible transmission route [48]. Interestingly, the earlier detection of viral H5N1/HP RNA in water followed by H7N1/HP and finally by H7N2/LP could mirror the initial ability of the virus to replicate in host cells, be shed, and thus, be more likely transmissible to naive birds. Drinking water should be particularly taken into account for quail and other game birds raised in outdoor operations, where AI viruses from wild birds could be introduced to the poultry flock. Furthermore, contamination of the environment by respiratory secretions and infected carcasses likely would result in indirect oral transmission of the virus. Although minor, cloacal shedding was consistently detected in HPAIV-infected quail, confirming that European quail might have functional binding receptors in both trachea and intestine, as already confirmed for both quail subspecies [24,25,49,50]. Besides, feathers could likely act as potential source for virus transmission in European quail, especially in recently dead birds susceptible to feather picking. To date, the relevance of feathers as a location for viral replication and potential origin of dissemination in HPAIV infection has been evidenced in certain bird species [4,51,52], but had not yet been demonstrated in quail.

The high degree of correlation between C-ELISA and HI results suggests that such tests seem to be equally sensitive and specific when assessing quail serological responses, as previously stated for Japanese quail [14]. Antibody response in HPAIV-inoculated quail started as early as 5 dpi, further confirming infection of the birds and an early humoral immune response. Seroconversion in H7N2/LP-infected quail at the end of the experiment proved effective infection not only among inoculated birds but also among contacts. In general, antibody titers in the present study were akin to those previously observed in AIV-infected Japanese quail [5,53,54] and gradually increased throughout the experiment, as already observed in H9N2 LPAIV-infected Japanese quail [54].

The high susceptibility of European quail to H7N1/HP and H5N1/HP would make this species a good sentinel of the presence of HPAIV in the environment, both in the wild or in semi-extensive farms. On the other hand, infected quail can shed a considerable amount of AIV before the appearance of overt clinical signs, death or both (around four days in the present experiment). Therefore, spreading disease into the wild by releasing apparently healthy farm-reared quail for hunting purposes could represent a substantial threat, even higher if assuming that this species could act as a mixing vessel like already stated for the Japanese quail. Furthermore, European quail may be considered sentinels (both for HPAIV and LPAIV) and reservoirs (for LPAIV), which is of special interest as most wild individuals are migratory [26]. The application of surveillance measures on quail flocks before and after release is of importance to avoid introduction of HPAIV, as well as other pathogens, in the natural ecosystem.

Current active AI surveillance activities include sampling of both OS and CS, as well as blood [55,56]. Passive surveillance of dead or moribund birds involves the same samples as for active surveillance (when possible) along with tissue collection through necropsy [55,56]. On the basis of our findings, OS could be used as a unique tool for successful virus detection in active AI surveillance programs in quail, as it has been assessed for other minor species in which pathogenesis is still poorly understood [4]. In addition, brain, pancreas, and heart specimens would be suitable in passive surveillance when HPAIV is suspected. Our results suggest that European quail, like Japanese quail, could play a key role in AI epidemiology because of the high susceptibility to HPAIV and the noteworthy spread of both HPAIV and LPAIV. Taking into account the similarities in viral dynamics between Japanese and European quail, the latter would also presumably have the capability to act as an intermediate host for avian/mammalian reassortant viruses, although further experiments are needed to address this issue. In addition, future studies comparing AI infection dynamics between Japanese and European quail by experimental infections with the same AIV strains would strengthen the present data. Altogether, our results underline the complexity of managing AI outbreaks when different susceptible species are involved.

## Competing interests

The authors declare that they have no competing interests.

## Author’s contributions

KB, NB, and FXA prepared the viruses used in this study. KB, RD, VG, JVA, AJC, and NM participated in the daily monitoring of the clinical signs and the sampling of the animals. KB, RD, VG, JVA, AJC, AR, and NM performed the necropsies and the tissue sampling. KB and NM carried out the histopathological examinations. KB carried out the RRT-PCR and the serology assays, together with FXA in the case of H5N1/HP samples. RD, NB, FXA, UH, and NM conceived the study and participated in its design and coordination. All authors read and approved the final manuscript.
